# Wheat Phenological Development and Growth Studies As Affected by Drought and Late Season High Temperature Stress under Arid Environment

**DOI:** 10.3389/fpls.2016.00795

**Published:** 2016-06-06

**Authors:** Muhammad Z. Ihsan, Fathy S. El-Nakhlawy, Saleh M. Ismail, Shah Fahad, Ihsanullah daur

**Affiliations:** ^1^Department of Arid Land Agriculture, Faculty of Meteorology, Environment and Arid Land Agriculture, King Abdulaziz UniversityJeddah, Saudi Arabia; ^2^College of Plant Science and Technology, Huazhong Agricultural UniversityWuhan, China

**Keywords:** wheat phenology, drought stress, arid environment, drip irrigation, Saudi Arabia

## Abstract

This study evaluates the potential for adaptability and tolerance of wheat genotypes (G) to an arid environment. We examined the influence of drought stress (DS) (100, 75, and 50% field capacity), planting times (PT) (16-November, 01-December, 16-December and 01-January), and G (Yocoro Rojo, FKAU-10, Faisalabad-08, and Galaxy L-7096) on phenological development, growth indices, grain yield, and water use efficiency of drip-irrigated wheat. Development measured at five phenological growth stages (GS) (tillering, jointing, booting, heading, and maturity) and growth indices 30, 45, 60, and 75 days after sowing (DAS) were also correlated with final grain yield. Tillering occurred earlier in DS plots, to a maximum of 31 days. Days to complete 50% heading and physiological crop maturity were the most susceptible GS that denoted 31–72% reduction in number of days to complete these GS at severe DS. Wheat G grown with severe DS had the shortest grain filling duration. Genotype Fsd-08 presented greater adaptability to studied arid climate and recorded 31, 35, and 38% longer grain filling period as compared with rest of the G at 100–50% field capacity respectively. December sowing mitigated the drought and delayed planting effects by producing superior growth and yield (2162 kg ha^−1^) at severe DS. Genotypes Fsd-08 and L-7096 attained the minimum plant height (36 cm) and the shortest growth cycle (76 days) for January planting with 50% field capacity. At severe DS leaf area index, dry matter accumulation, crop growth rate and net assimilation rate were decreased by 67, 57, 34, and 38% as compared to non-stressed plots. Genotypes Fsd-08 and F-10 were the superior ones and secured 14–17% higher grain yield than genotype YR for severely stressed plots. The correlation between crop growth indices and grain yield depicted the highest value (0.58–0.71) at 60–75 DAS. So the major contribution of these growth indices toward grain yield was at the start of reproductive phase. It's clear that booting and grain filling are the most sensitive GS that are severely affected by both drought and delay in planting.

## Introduction

Drought and temperature extremes are the major abiotic constraints to cereal production worldwide, particularly in rain-fed dry agriculture systems (Venkateswarlu and Shanker, 2012). The western region of Saudi Arabia is classified into rain fed dry area by Koppen's classification scheme, due to its arid land features. Unlike other regions of the peninsula, the western region retains extreme hot temperature (over 30°C) even in the winter (Aburas et al., 2011). Under changing environmental features globally and increasing world population, crop production and water resources are declining day by day, along with the increased frequency of extreme temperature fluctuations. It is therefore, imperative to develop heat and drought tolerant wheat genotypes (G) to match the ever increasing demand for food supply (Buck et al., 2007; Hossain et al., 2013). Development of new wheat G requires huge capital and sufficient time. However, testing adaptability of already developed exotic G may serve the purpose for short term solution. It is hypothesized that selected exotic G may outcompete the local G and can replace them based on their performance under local arid land conditions.

Wheat (*Triticum aestivum* L.) is one of the earliest plants cultivated by mankind and originated from the Levant region of the near East and Ethopian Highlands (Haider, 2013). At present it is second major cereal crop after rice and ahead of maize with production of 735 million tons annually (FAO, FAOSTAT, 2011; USDA, 2015). Wheat is a major food staple in the human diet with a contribution of 19% in world food energy and 21% in total protein intake, which makes it the highest by any single crop (D'souza and Jolliffe, 2012). Currently, wheat is an essential staple food for ~2 billion people and documented as severely affected by drought and heat stresses (Turral et al., 2011). More than one third of the world's total cultivated area is affected by drought stress (DS). Within that area 33% (99 million hectares) belongs to developing countries and 25% (60 million hectares) belongs to developed nations (Rijsberman, 2006). A noteworthy (at least 50%) increase in production of major cereal crops like rice, wheat and maize is required to meet the needs for the projected population by 2050 (Godfray et al., 2010; Fahad et al., 2015a,b, 2016). The Kingdom of Saudi Arabia (KSA) has a total area of 224 million hectares, and 21.7% (49 million hectares) of it is potentially suitable for cultivation (FAO, FAOSTAT, 2011). Currently, only 2.2% (4.9 million hectares) is cultivated due to unavailability of fresh water resources, high temperature and drought extremes (FAO, FAOSTAT, 2011).

Drought is a non-uniform phenomenon that negatively influences plant growth, morphology, physiology and yield depending upon crop developmental stage, time, and severity of stress (Farooq et al., 2009; Ahmad and Prasad, 2011). Drought tolerance is exceedingly complex and multigenic trait in plants that implies a combination of genetic, physiological, and biochemical mechanisms (Farooq et al., 2009). Crops at various growth stages (GS) require variable level of moisture and temperature for optimum growth. The plants requirement for water, nutrient, and CO_2_ increases with every next crop phenological GS starting from germination. This increased uptake is used to fulfill energy requirements for higher rate of evapotranspiration, photosynthesis, respiration, and development (Blum, 2011).

Plants have limited nutrient uptake capacity and photosynthetic efficiency under heat and drought stress. These stresses can also reduce organ size (leaf, tiller, and spikes) and growth period for various development stages (tillering, jointing, booting, heading, anthesis, and grain filling) (Hossain et al., 2013). Plant sensitivity to drought and high temperature result in disturbed metabolic processes coupled with shorter plant life cycle (Tuteja and Sarvajeet, 2012) and consequently lowered plant biomass accumulation and grain yield (Hasanuzzaman et al., 2013).

Global mean temperature has risen at a rate of 0.3°C per decade during the twentieth-century (Jones et al., 1999) and is expected to reach 1 and 3°C above the current value by 2025 and 2100 respectively, resulting in more severe global climatic changes in near future. This projection is generating apprehension among researchers, as high temperature stress has known influences on the life processes of organisms, acting directly or through the variation of surrounding environmental components. Therefore, selection of heat and drought tolerant wheat G is a priority to manage the adverse effects of these stresses (Alghabari et al., 2014). Development of drought and heat tolerant G require time without an assurance of their desired performance under specified rain-fed conditions. However, screening of already available dwarf (*Rht*) G for their adaptability to arid land conditions may be achievable in a shorter period of time. Therefore, the present study was carried out: (1) to evaluate the adaptability of four wheat G to arid land environment of Saudi Arabia, (2) to examine the response of wheat phenology and growth indices to different levels of DS, (3) to adjust the planting date of newly screened G to local climate under rain fed drip irrigation system, and (4) to improve the understanding regarding the role of each GS toward grain development under stressed and non-stressed conditions. The four wheat G used in this experiment were selected on their germination index and seedling establishment capacity under DS in a lab experiment, among a pool of fifty G. The result of this study is expected to improve our knowledge on wheat phenological development, irrigation use efficiency and adaptability of potential of exotic G to rain fed conditions. The result will help to improve water conservation through efficient or deficit irrigation approach based on the sensitivity of the phenological GS and their contribution toward final grain yield. This research will also help small farmers of rain fed areas to conserve moisture by efficient use at the most critical developmental stages of plant and by optimizing the planting time of exotic G to coincide with expected rainfall or soil water availability. Based on the performance of exotic G, they may be included in the breeding program for the development of drought tolerant G for rain fed areas.

## Materials and methods

### Site description

**Figure 1:**
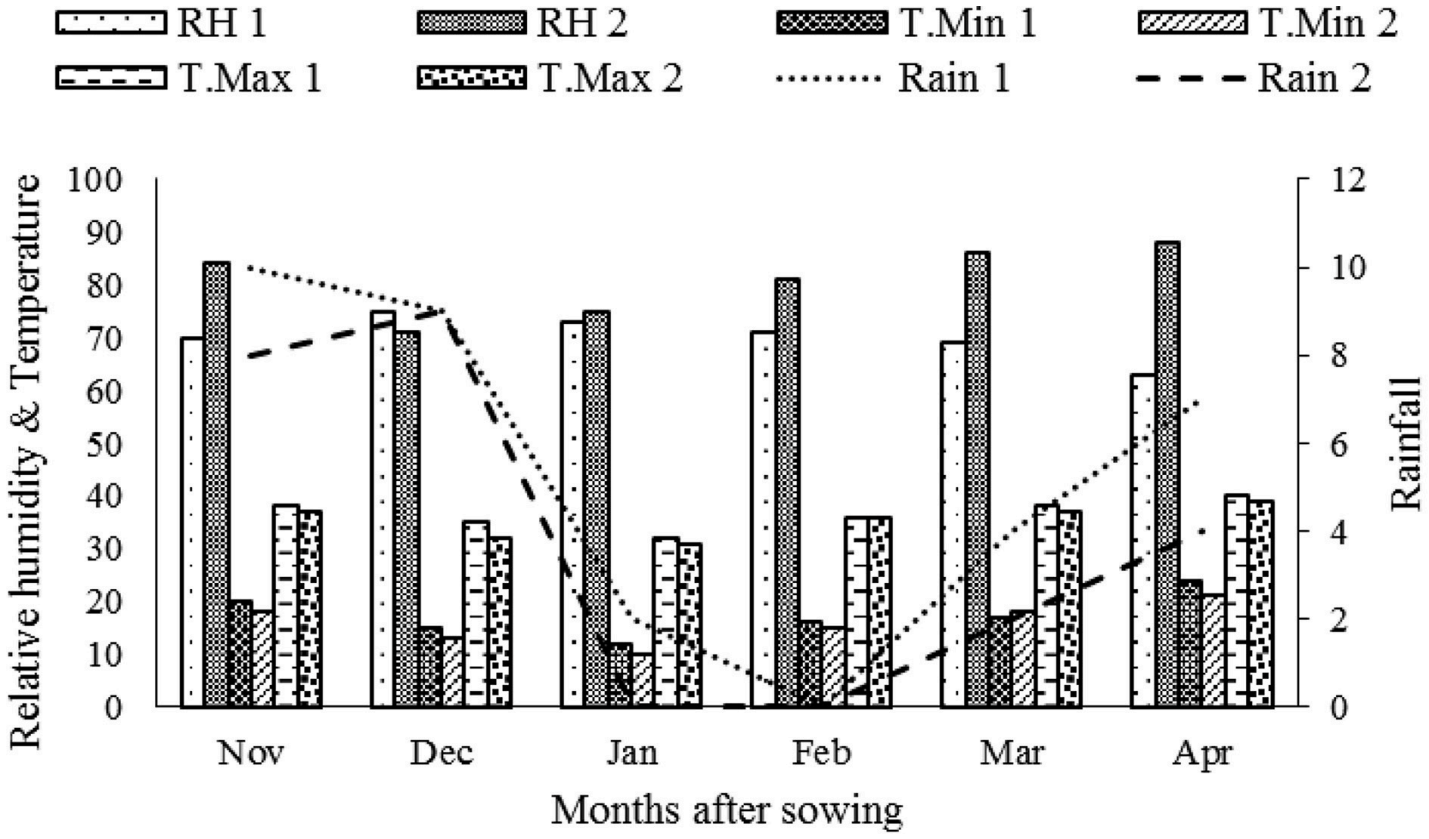
**Averaged meteorological features of experimental site**. RH: relative humidity (%), T. max: maximum temperature (°C), T. min: minimum temperature (°C), and rainfall (mm). the value 1 represent last 10 year average data (2004–13) and 2 represent the experimental year data.

The experiment was conducted at the Agricultural Experimental Station of King Abdulaziz University located at Hada-Al Sham, 110 km North East of Jeddah, KSA (21° 48′ 3″ N, 39° 43′ 25″ E) during 2013–14 growing season. Hada Al Sham is considered as an extreme arid region with harsh climatic features. Meteorological data of the experimental site during the study period are presented in Figure 1. The soil for experimental site was classified as sandy loam with a bulk density of 1.67 g cm^−3^, electrical conductivity (EC) of the soil and irrigation water 1.63 dS^−1^ and 4.61 dS^−1^ and pH 8.35 respectively. The soil was very poor in organic matter (0.58%) and available nutrients (N: 0.37 mg/kg, P: 0.14 mg/kg and K: 2.9 mg/kg).

### Plant material

Four wheat (*T. aestivum* L.) genotypes (G), Yocoro Rojo (YR), Faisalabad 2008 (Fsd-08), FKAU-10 (F-10), and Galaxy L-7096 (L-7096) were grown under different water regimes and planting dates (PD) under arid land conditions to study the phenological variations. Genotypes Fsd-08 and L-7096 were procured from Agronomic Section, Ayub Agriculture Research Institute (AARI), Faisalabad, Pakistan. Genotype F-10 was collected from the Field Crops Lab, Department of Arid Land Agriculture, King Abdulaziz University, Jeddah, Saudi Arabia. Genotype YR was commercially recommended and commonly cultivated variety in the Saudi Arabia.

### Crop husbandry

The selected experimental site was plowed 1 month prior to planting of seed. Two weeks later, soil was again plowed and was followed by planking. Fertilizer was used at a rate of 200:150:100 kg N:P:K ha^−1^. The fertilizer was a 20:20:10 (NPK) mixture, diammonium phosphate (18% N, 46% P), and urea (46% N). During the time of seed bed preparation all of the P and K but only one-third of N were applied. The remaining two-thirds of N was top dressed at tillering and booting stages in equal amounts. Green foxtail (*Setaria viridis*) and desert horse purslane (*Trianthema portulacastrum*) were the major weeds of the experimental site. Manual pulling/weeding was executed at 15 and 30 days after sowing (DAS) to keep weeds below the economic threshold level. All other agronomic practices were similar between treated and non-treated plots.

### Irrigation system installation and water supply

After leveling the soil of the experimental site, surface drip irrigation system was installed. The distance between drippers was 30 cm. The type of the dripper line was *RAIN BIRD LD- 06- 12-1000 Landscape drip 0.6 G/h @12"*. The downstream end of each dripper line was connected to a manifold for convenient flushing. Inlet pressure on each tape was ~1.5 bars. The system used a 125-micron disk filter. Two containers with a capacity of 6000 L each were installed for the storage of water. These containers were routinely filled via a main irrigation network, and water supply was automatically controlled by water electronic module (WEM). In WEM technology, the water requirement of the growing plants for each treatment was calculated based on the available soil moisture of the root zone. The field capacity (FC) was measured at 30 cm soil depth using the pressure plate method in the laboratory. Tensions of 10–20, 30, and 40–50 cb were used to achieve 100, 75, and 50% FC, respectively. The WEM was programmed with these user-input soil water tension, and the irrigation system automatically supplied the required amount of water based on the daily shortage of the measured soil moisture tension. A detailed description for WEM technology is presented in Ismail et al. (2013). Daily irrigation of 2.8 mm (10 min) for 1 week was applied prior to planting, to attain soil moisture content at 100% FC level. The FC of 100, 75, and 50% were considered as non-stressed, mild stressed, and severely stressed, respectively.

### Experimental design and treatments

The experiment was laid out in a randomized complete block design (RCBD) with split-split plot arrangement, having a net plot size of 3 × 2 m with four replications. Drought stress (100, 75, and 50% FC) was the main plot treatment. Sub-plot treatments consisted of four PD: 16 November (PD1), 01 December (PD2), 16 December (PD3), and 01 January (PD4). Sub-sub plot treatments constituted of the four wheat G (YR, Fsd-08, F-10, and L-7096). The total numbers of treatments were 48, while the numbers of experimental units were 192. All experimental units were kept at 100% FC for 2 weeks after sowing, to provide sufficient time for crop establishment prior to stress treatments application.

Line sowing of wheat was performed manually maintaining the line to line distance at 20 cm and line to drip distance at 10 cm. Seed was applied at a rate of 180 kg ha^−1^ due to lower seedling establishment rate under the field conditions. The total amount of applied water for each planting date and DS level is presented in Table 1.

**Table 1:** **Total amount of applied water (mm) at each planting date to maintain required field capacities during crop growth cycle (2013–14)**.

**Date**	**PD1**			**PD2**			**PD3**			**PD4**		
**Field capacities (FC %)**												
	**100%**	**75%**	**50%**	**100%**	**75%**	**50%**	**100%**	**75%**	**50%**	**100%**	**75%**	**50%**
10-20 Nov	19.60	19.60	19.60									
21-30 Nov	14.00	8.40	07.00									
01-10 Dec	14.00	8.40	07.00	28.00	28.00	28.00						
11-20 Dec	19.60	14.00	10.00	11.20	08.40	05.60	19.60	19.60	19.60			
21-31 Dec	30.80	22.40	16.80	18.48	15.40	09.24	12.32	09.24	06.16			
01-10 Jan	39.20	30.80	19.00	16.60	14.00	08.30	14.00	11.20	07.00	28.00	28.00	28.00
11-20 Jan	39.20	30.80	19.60	22.40	16.80	11.40	14.00	11.20	07.00	14.00	11.20	07.00
21-31Jan	53.20	42.00	28.00	43.12	33.88	21.56	36.40	25.20	19.60	15.40	12.32	9.24
01-10 Feb	50.40	39.20	25.20	50.40	36.40	25.20	39.20	30.80	19.60	28.00	22.40	14.00
11-20 Feb	53.20	39.20	–	47.60	36.40	25.20	53.20	39.90	26.60	39.20	30.80	19.60
21-28 Feb	14.00	–	–	38.08	29.12	20.16	47.07	35.86	24.64	33.60	24.64	17.92
01-10 Mar				61.60	47.60	–	64.40	47.60	33.60	64.40	50.40	33.60
11-20 Mar				56.00	21.00	–	67.20	50.40	16.60	67.20	50.40	33.60
21-31 Mar				21.00	–	–	38.50	–	–	56.00	21.00	14.00
01-10 Apr										10.08	–	–
Total (mm)	347.20	254.80	152.80	414.48	287.00	154.66	405.89	281.00	180.40	355.88	251.16	176.96

*PD1, 16 November; PD2, 01 December; PD3, 16 December and PD4, 01 January are planting times for genotypes*.

### Data collection

Average plant height (cm) of 10 randomly selected plants from each plot was recorded using measuring tape at five phenological GS viz., tillering, jointing, booting, heading, and maturity. These GS were identified by using Zadoks Cereal Growth Stages key. Growing degree days to complete these phenological GS were also recorded for each DS, PD, and G. Plant fresh (g) and dry weight (g) from each plot at two randomly selected sites (0.5 × 0.5 m) were recorded and converted to g m^−2^. Leaf area index (LAI) was measured through LAI-2200C Plant Canopy Analyzer. Plant fresh and dry weight along with LAI were used to measure dry matter accumulation (DMA; g m^−2^), leaf area duration (LAD; days), crop growth rate (CGR; g m^−2^ d^−1^), and net assimilation rate (NAR- g m^−2^ d^−1^) at 30, 45, 60, 75, and 90 DAS (Hunt, 1982; Beadle, 1985). At physiological crop maturity, an area of 1 × 1 m was manually harvested and threshed from each sub-sub plot. The grain weight was measured (g m^−2^) and adjusted at 12% moisture content. This adjusted weight was converted to kg ha^−1^. The water use efficiency was measured by dividing grain yield to total amount of applied water from sowing to maturity and presented as kg ha^−1^ mm^−1^. Stress tolerance index was calculated by using equation defined by Fernandez (1992).

### Statistical analysis

For phenological GS, crop growth indices, grain yield and stress indices the data was statistically analyzed using Fisher's analysis of variance technique (*p* ≤ 0.05) in SAS 8.1 (Statistix 8.1, Analytical software, Statistix; Tallahassee, FL, USA, 1985–2003). Treatment means (G, DS and PD) were separated on the bases of least significant difference (LSD) at *p* ≤ 0.05 probability level (Steel et al., 1997). Simple linear regression analyses were drawn for dependent variable (grain yield) and independent variables (LAI, DMA, and CGR) at 30, 45, 60, and 75 DAS to estimate the contribution of each growth indices and growth stage toward final grain development.

## Results

### Wheat phenology

**Figure 2:**
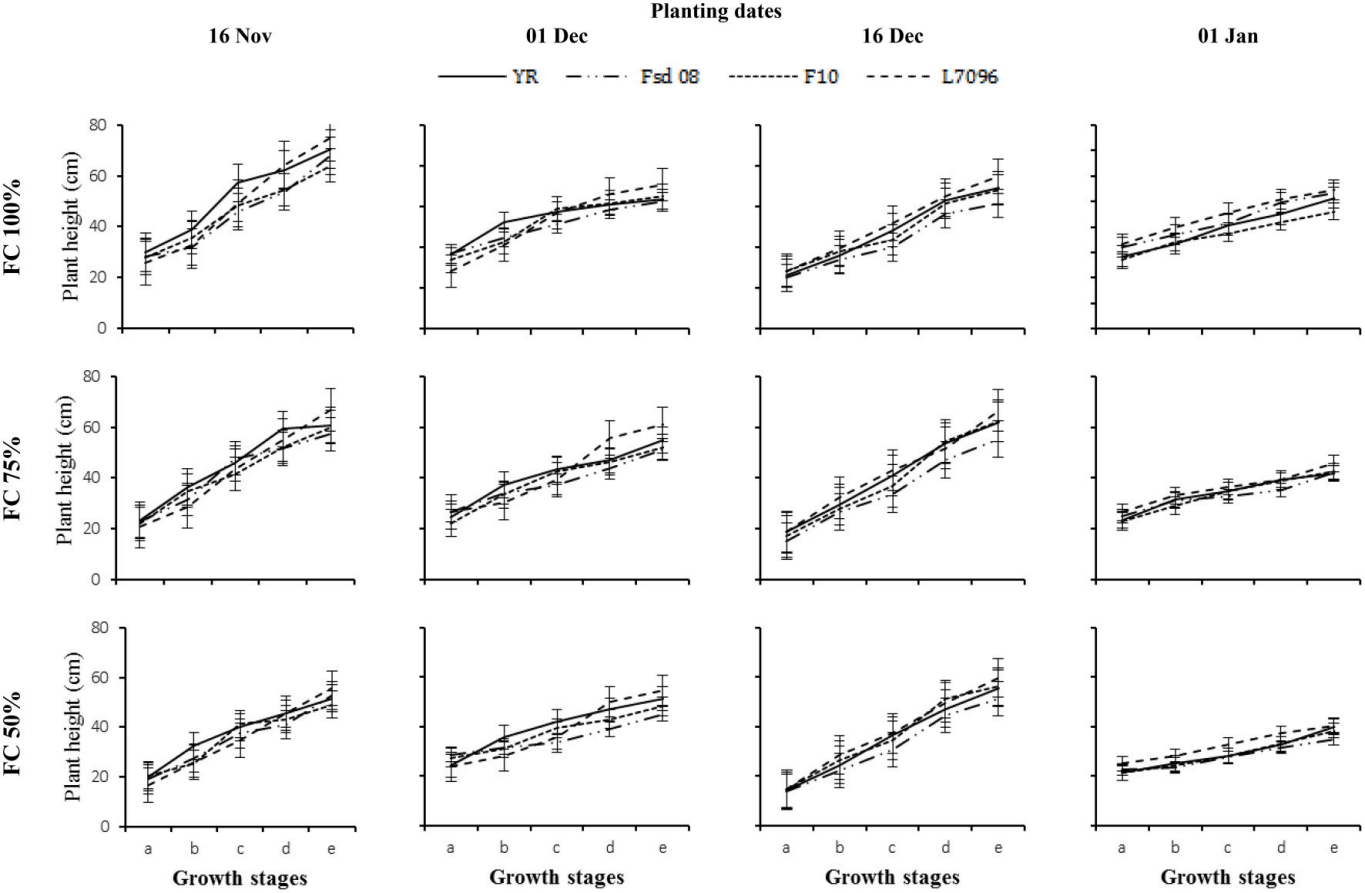
**Plant height (cm) of four wheat genotypes (G), viz., Yocoro Rojo (YR), Faisalabad 2008 (Fsd-08), FKAU-10 (F-10), and Galaxy L-7096 (L-7096) at three field capacities (FC) (100, 75, and 50%) and four planting dates (PD) (16 November; 01 December; 16 December and 01 January)**. Wheat Phenological growth stages i.e., tillering, jointing, booting, heading and maturity are denoted by a, b, c, d, and e, respectively. Vertical points are standard error bars representing statistically significant level of difference of each growth stage for studied G, PD and FC levels. Straight line, dotted dashed line, dotted line and dashed line illustrates YR, Fsd-08, F-10, and L-7096 genotypes. Least significant difference (LSD) values among treatment means were 2.69, 2.56, 3.27, 2.79, and 3.11 for tillering, jointing, booting, heading, and maturity stages, respectively.

Statistical evaluation of the recorded data for days to complete wheat phenological GS (tillering, jointing, booting, heading and maturity) and plant height at these GS presented pronounced variations under the influence of various DS, PD, and G. The main effect of these three factors were significant (*p* ≤ 0.05) except, number of days required to complete tillering stage for DS; plant height and days taken to complete tillering and jointing stages for PD and plant height at tillering stage for G (Table 2). Two way interaction of G to PD and DS were highly significant (*p* ≤ 0.01) for most of the studied GS. Three way interactions of DS × PD × G was significant (*p* ≤ 0.05) for plant height at jointing and heading stages, while number of days to complete these GS were also significant (*p* ≤ 0.05) for all stages except jointing. Results depicted that DS significantly reduced the number of days to complete the studied phenological GS; nevertheless, such damaging effects of DS were minimized when wheat was planted early in the growing season. Differences among G were also significant (*p* ≤ 0.05) under the influence of DS, and PD and the negative effect of DS was more severe on L-7096 and YR as compared with those recorded on Fsd-08 and F-10. Regardless of different DS and PD, the maximum plant height was recorded for L-7096 while prolonged growth cycle was observed in Fsd-08 (Figure 2).

**Table 2:** **Statistical analysis of crop phenological growth stages and crop growth indices recorded at each growth stage and 15 days interval**.

**SOV**	***d.f***	**Plant height**					**Days to complete phenological GS**					**Leaf area index**					**Dry matter accumulation**				
		**Phenological growth stages**										**Days after sowing**									
		**Till**	**Jont**	**Boot**	**Head**	**Matu**	**Till**	**Jont**	**Boot**	**Head**	**Matu**	**30**	**45**	**60**	**75**	**90**	**30**	**45**	**60**	**75**	**90**
R	3	ns	ns	ns	ns	ns	ns	ns	ns	ns	ns	ns	ns	ns	ns	ns	ns	ns	ns	ns	ns
FC	2	^*^	^**^	^**^	^*^	^**^	ns	^*^	^**^	^**^	^**^	ns	^*^	^*^	^**^	^**^	^*^	ns	^*^	^**^	^**^
Error a	6	–	–	–	–	–	–	–	–	–	–	–	–	–	–	–	–	–	–	–	–
PD	3	ns	ns	^*^	^**^	^**^	ns	ns	^**^	^*^	^**^	ns	ns	^*^	ns	^*^	ns	^*^	^**^	^*^	^**^
FC^*^PD	6	^*^	^**^	^**^	^**^	ns	^**^	ns	^*^	ns	^**^	ns	^*^	ns	^*^	^**^	ns	ns	^**^	^**^	^*^
Error b	27	–	–	–	–	–	–	–	–	–	–	–	–	–	–	–	–	–	–	–	–
G	3	ns	^*^	^**^	^*^	^**^	^*^	^*^	^**^	^*^	^**^	ns	^*^	^**^	^**^	ns	^*^	^*^	^*^	^**^	^**^
FC^*^G	6	^*^	ns	^**^	^**^	ns	^*^	ns	^**^	^**^	^**^	^*^	^**^	^*^	^**^	^*^	ns	ns	^**^	^*^	^*^
PD^*^G	9	ns	^*^	ns	^**^	^*^	^**^	^**^	^**^	^**^	^**^	ns	^*^	^**^	^**^	^*^	^*^	^**^	^**^	^**^	^**^
FC^*^PD^*^G	18	ns	^*^	ns	^*^	ns	^*^	ns	^**^	^*^	^**^	^**^	ns	^**^	^**^	ns	ns	^*^	ns	^**^	^*^
Error c	108	–	–	–	–	–	–	–	–	–	–	–	–	–	–	–	–	–	–	–	–
Total	191	–	–	–	–	–	–	–	–	–	–	–	–	–	–	–	–	–	–	–	–
LSD	–	2.69	2.56	3.27	2.79	3.11	2.01	1.97	2.66	3.30	5.16	0.20	0.22	0.29	0.28	0.19	2.28	10.45	11.16	11.22	4.87
CV	–	7.93	5.63	5.67	4.02	6.93	6.20	3.99	4.30	4.19	3.89	7.99	7.79	4.06	5.16	7.93	3.82	6.10	2.27	1.32	2.32

ns, non-significant;

* , significant at p ≤ 0.05;

** *, significant at p ≤ 0.01; SOV, source of variation; d.f, degree of freedom; R, replication; FC, field capacity; PD, planting date; G, genotype; LSD, least significant difference; CV, coefficient of variation; GS, growth stages; Till, tillering; Jont, jointing; Boot, booting; Head, heading; Matu, maturity*.

When averaged across the four PD, the mild and severe DS reduced the plant height of YR by 63–79%, Fsd-08 by 54–88%, F-10 by 62–77%, and L-7096 by 66–84%, as compared with non-stressed plots respectively. Likewise, delay in planting reduced the plant height; wheat sown in January was 34 and 15% shorter than November (16–30 November) planted wheat respectively (Figure 2). All G sown in mid-November described non-significant variations to applied DS levels. Genotypes Fsd-08 and F-10 proved more tolerant to high temperature, and performed better for late plantation.

Early tillering was prominent in plots subjected to DS (50 or 75% FC) irrespective of PD and G. On an average, 20–31 days were taken to complete tillering stage at 100% FC that was significantly reduced at 75% FC and 50% FC. Days to complete 50% heading and physiological crop maturity were the most susceptible to DS. A 75–50% FC resulted in a reduction of 31–72% in number of days to complete these GS as compared to 100% FC (Table 4). Wheat planted in November took maximum time to complete vegetative and reproductive GS as compared with December or January planting. January planting reported the shortest crop cycle. All the G sown on the first week of December followed a steady growth behavior with elongated time span to complete each of the crop GS. At 100% FC, the wheat G followed a gradual transition from vegetative to reproductive stage. Nevertheless, plants grown under 75 or 50% FC had a short grain filling period as indicated by the sharpness of the growth curve entering from vegetative to reproductive phase. Genotypes Fsd-08 and F-10 were statistically similar to complete tillering, jointing, and booting stages, but their responses during the reproductive phase was changed. Genotype Fsd-08 demonstrated greater adaptability to studied arid climate and recorded 31, 35, and 38% longer grain filling period as compared with F-10, YR, and L-7096 at 100% FC to 50% FC, respectively. Genotype L-7096 sown in the first week of January at 50% FC took the minimum days (76 days) to complete physiological crop maturity.

### Wheat growth indices

Crop growth indices viz., leaf area index (LAI), leaf area duration (LAD; days), dry matter accumulation (DMA; g m^−2^), crop growth rate (CGR; g m^−2^ d^−1^), and net assimilation rate (NAR; g m^−2^ d^−1^) were recorded at 30, 45, 60, 75, and 90 DAS for all G under different PD and DS (Tables 2, 3). Statistical analysis of the recorded data depicted significant (*p* ≤ 0.05) effects of DS and G while non-significant (*p* ≥ 0.05) effect of PD was recorded for most of the studied growth indices and their recording intervals. Interaction of DS × PD was significant (*p* ≤ 0.05) for LAI, LAD, CGR, and NAR at the start of reproductive phases except for NAR at maturity, while non-significant effect at tillering and jointing stages were observed for these growth indices except LAD that was significant at 30 and 45 DAS. Interaction of G × DS × PD was variable and most of the GS resulted in significant interaction, except for NAR at maturity and LAI, LAD, and CGR at tillering stage. Three factors DS × PD × G interaction was significant (*p* ≤ 0.05) for DMA at 45 and 90 DAS; LAD for 60 and 75 DAS; and CGR for 45 DAS. Significant (*p* ≤ 0.01) effect of FC × PD × G interaction was recorded for LAI at 30, 60, and 75 DAS; for DMA at 75 DAS; for LAI and CGR at reproductive GS. Non-significant effect for three way interaction was observed for LAI and DMA at 30-45 DAS while for CGR and NAR at 75-90 DAS (Table 3).

**Table 3:** **Statistical analysis of crop growth indices recorded at 15 days interval from tillering to maturity**.

**SOV**	***d.f***	**Leaf area duration**					**Crop growth rate**				**Net assimilation rate**				
		**Days after sowing**													
		**30**	**45**	**60**	**75**	**90**	**30**	**45**	**60**	**75**	**30**	**45**	**60**	**75**	**90**
R	3	ns	ns	ns	Ns	Ns	ns	ns	ns	ns	ns	ns	ns	Ns	ns
FC	2	^*^	^*^	^**^	^**^	^**^	ns	^**^	^*^	^**^	^*^	^**^	^**^	^**^	ns
Error a	6	–	–	–	–	–	–	–	–	–	–	–	–	–	–
PD	3	ns	ns	^**^	^**^	^**^	ns	^**^	^**^	^**^	^*^	^**^	^**^	^**^	^*^
FC^*^PD	6	^*^	^*^	ns	^*^	^**^	ns	^*^	^**^	^**^	^*^	ns	^*^	^**^	ns
Error b	27	–	–	–	–	–	–	–	–	–	–	–	–	–	–
G	3	ns	^**^	^**^	^*^	^**^	^*^	^**^	^**^	ns	^*^	^*^	^**^	^**^	ns
FC^*^G	6	ns	^**^	ns	^*^	^**^	ns	^*^	^**^	^**^	^**^	^*^	^*^	^**^	^*^
PD^*^G	9	^*^	^**^	^*^	^**^	^*^	^**^	^*^	ns	^**^	ns	^*^	^**^	^*^	ns
FC^*^PD^*^G	18	ns	ns	^*^	^*^	^**^	ns	^*^	^**^	ns	^**^	^**^	^**^	^**^	ns
Error c	108	–	–	–	–	–	–	–	–	–	–	–	–	–	–
Total	191	–	–	–	–	–	–	–	–	–	–	–	–	–	–
LSD	–	0.20	0.31	0.29	0.28	0.19	0.49	0.38	0.81	0.29	0.04	0.09	0.19	0.16	0.04
CV	–	7.99	7.78	4.12	5.16	7.93	7.07	1.82	3.05	6.14	2.38	2.93	1.35	4.81	2.46

ns, non-significant;

* , significant at p ≤ 0.05;

** *, significant at p ≤ 0.01; SOV, source of variation; d.f, degree of freedom; R, replication; FC, field capacity; PD, planting date; G, genotype; LSD, least significant difference; CV, coefficient of variation*.

**Table 4:** **Genotypic variation for days taken to complete each phenological growth stage as affected by water stress and planting time under arid land conditions**.

**Field capacity (%)**	**Planting dates**	**Yocoro Rojo**					**Faisalabad 08**				
		**T**	**J**	**B**	**H**	**M**	**T**	**J**	**B**	**H**	**M**
		**Days to complete phenological growth stages**									
100	PD1	24.01	41.25	47.25	63.85	98.40	26.25	46.51	52.25	73.12	96.71
	PD2	27.25	32.51	37.75	58.25	105.01	20.25	34.75	42.25	65.25	116.75
	PD3	25.00	30.75	49.25	57.04	113.01	24.00	35.75	53.25	67.00	118.75
	PD4	29.75	38.25	43.75	59.25	91.75	28.01	41.51	47.25	66.75	98.08
75	PD1	22.75	41.02	47.75	53.50	85.32	21.25	43.04	57.75	61.25	91.15
	PD2	23.02	28.75	35.03	54.01	101.75	19.75	34.75	39.25	60.25	111.01
	PD3	22.51	27.73	42.04	54.02	98.25	19.05	31.50	49.51	57.25	104.00
	PD4	24.03	37.75	42.25	49.25	83.25	22.25	39.75	53.06	57.25	87.75
50	PD1	23.51	37.03	44.00	47.00	83.34	21.25	40.72	52.01	58.5	87.41
	PD2	21.25	26.75	33.25	51.07	94.25	18.75	31.52	36.50	57.75	105.51
	PD3	18.50	24.75	34.75	45.09	88.06	18.04	26.06	37.25	53.50	97.75
	PD4	19.75	34.07	39.03	42.02	79.87	21.25	37.51	43.75	53.25	85.25
FC^*^PD^*^G		^*^	^*^	^*^	^**^	^**^	ns	^*^	^*^	^*^	^*^
LSD (*p ≤ 0.05*)		1.78	4.52	2.33	5.52	4.78	–	2.10	3.76	3.56	3.87
**Field capacity (%)**	**Planting dates**	**F-10**					**L-7096**				
		**T**	**J**	**B**	**H**	**M**	**T**	**J**	**B**	**H**	**M**
		**Days to complete phenological growth stages**									
100	PD1	28.51	45.25	57.25	72.71	93.02	31.01	42.51	46.52	63.05	95.56
	PD2	22.00	32.75	37.25	60.51	124.25	25.50	35.04	41.75	65.75	109.50
	PD3	26.03	32.25	54.75	63.25	103.06	25.75	34.00	47.50	59.05	97.04
	PD4	29.25	42.25	52.04	66.25	90.51	27.25	39.75	41.75	57.75	87.75
75	PD1	27.05	44.51	52.25	57.52	86.18	27.52	42.25	47.25	51.55	88.18
	PD2	21.75	30.75	36.00	56.75	110.75	24.51	32.51	38.51	58.07	104.25
	PD3	22.01	28.04	47.75	54.54	93.51	21.03	29.75	44.54	52.26	92.75
	PD4	25.00	40.51	46.52	53.01	85.50	23.04	39.25	41.50	47.54	82.54
50	PD1	23.50	39.52	44.75	50.25	82.74	21.75	38.25	45.54	50.43	79.42
	PD2	17.07	28.54	32.75	52.00	96.25	19.04	29.04	37.65	54.75	86.75
	PD3	17.51	24.03	41.75	48.25	92.25	18.03	25.04	39.56	43.54	86.50
	PD4	23.25	36.09	40.04	45.25	81.04	24.25	35.04	40.00	45.25	76.25
FC^*^PD^*^G		ns	^*^	^*^	^*^	^**^	ns	^*^	^*^	^*^	^*^
LSD (*p ≤ 0.05*)		–	2.11	4.16	7.33	4.32	–	3.43	3.87	4.54	3.22

ns, non-significant;

* , significant at p ≤ 0.05;

** *, significant at p ≤ 0.01; FC, field capacity; PD, planting date; G, genotype; T, tillering; J, jointing; B, booting; H, heading; LSD, least significant difference*.

**Figure 3:**
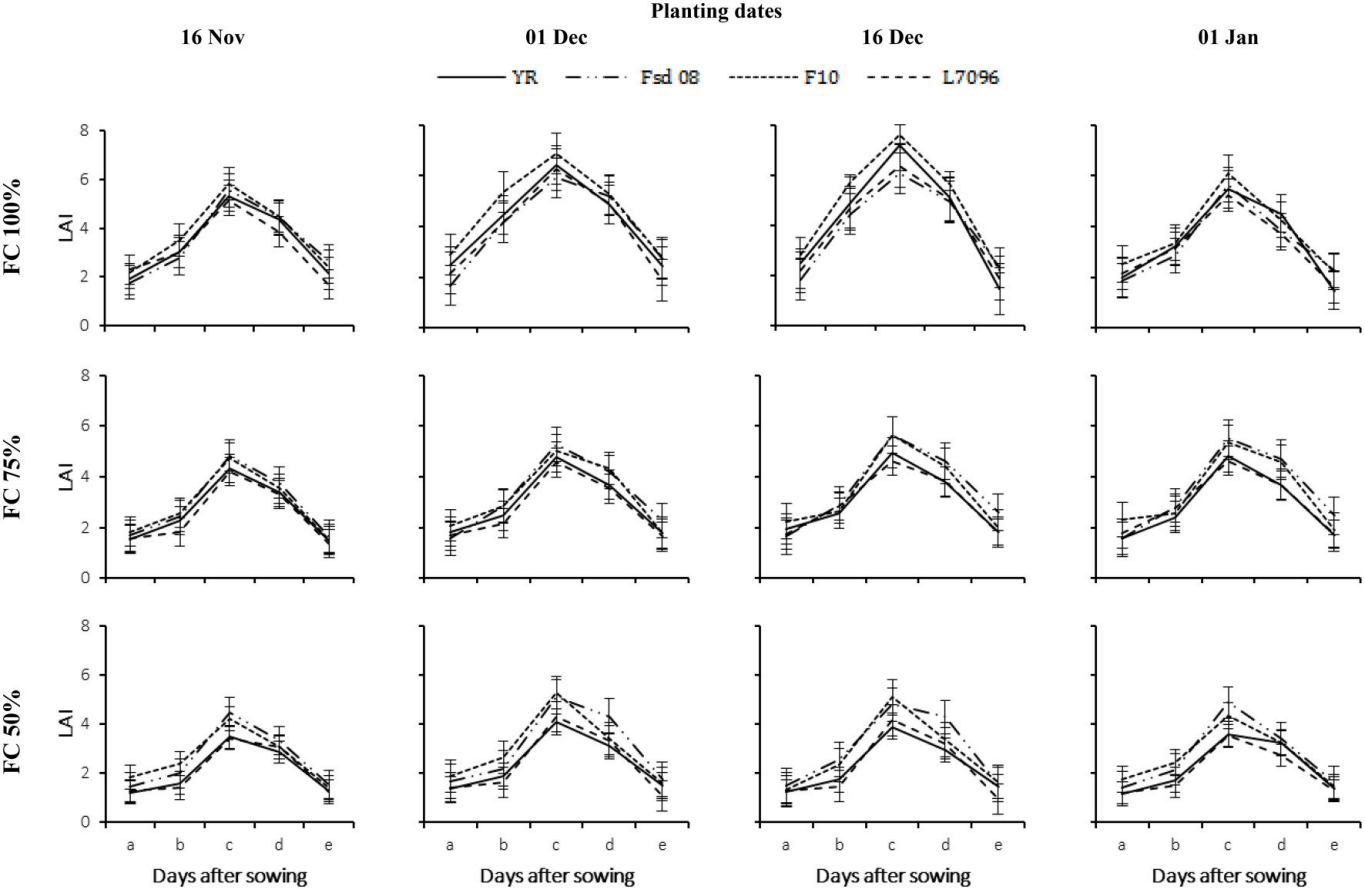
**Leaf area index (LAI) of four wheat genotypes (G), viz., Yocoro Rojo (YR), Faisalabad 2008 (Fsd-08), FKAU-10 (F-10), and Galaxy L-7096 (L-7096) at three field capacities (FC) (100, 75, and 50%) and four planting dates (PD) (16 November; 01 December; 16 December, and 01 January) recorded at 15 days interval**. LAI was recorded from 4th weeks of sowing (2nd week of drought stress treatment application) at 30, 45, 60, 75, and 90 days after sowing (DAS) and denoted by a, b, c, d, and e respectively. Vertical points are standard error bars representing statistically significant level of difference for LAI recorded at 15 days interval for studied G, PD, and FC levels. Straight line, dotted dashed line, dotted line, and dashed line illustrate YR, Fsd-08, F-10, and L-7096 genotypes. Least significant difference (LSD) values among treatment means were 0.20, 0.22, 0.29, 0.28, and 0.19 for a, b, c, d, and e respectively.

**Figure 4:**
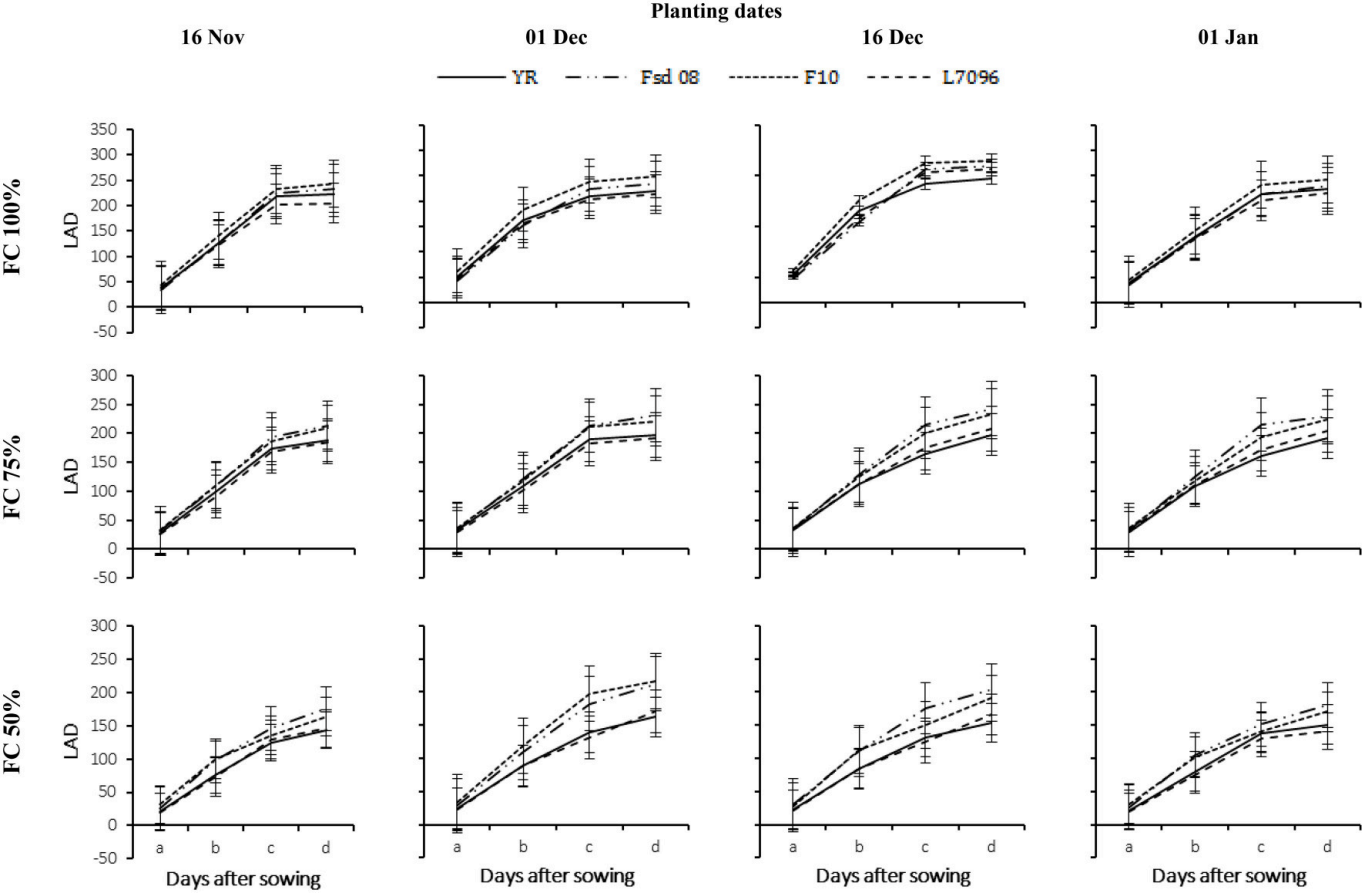
**Leaf area duration (LAD) of four wheat genotypes (G), viz., Yocoro Rojo (YR), Faisalabad 2008 (Fsd-08), FKAU-10 (F-10), and Galaxy L-7096 (L-7096) at three field capacities (FC) (100, 75, and 50%) and four planting dates (PD) (16 November; 01 December; 16 December, and 01 January) recorded at 15 days interval**. LAD was recorded from 4th weeks of sowing (2nd week of drought stress treatment application) at 30–45, 45–60, 60–75, and 75–90 days after sowing (DAS) denoted by a, b, c, and d respectively. Vertical points are standard error bars representing statistically significant level of difference for LAD recorded at 15 days interval for studied G, PD, and FC levels. Straight line, dotted dashed line, dotted line, and dashed line illustrate YR, Fsd-08, F-10, and L-7096 genotypes. Least significant difference (LSD) values among treatment means were 0.20, 0.31, 0.29, and 0.28 for a, b, c, and d respectively.

**Figure 5:**
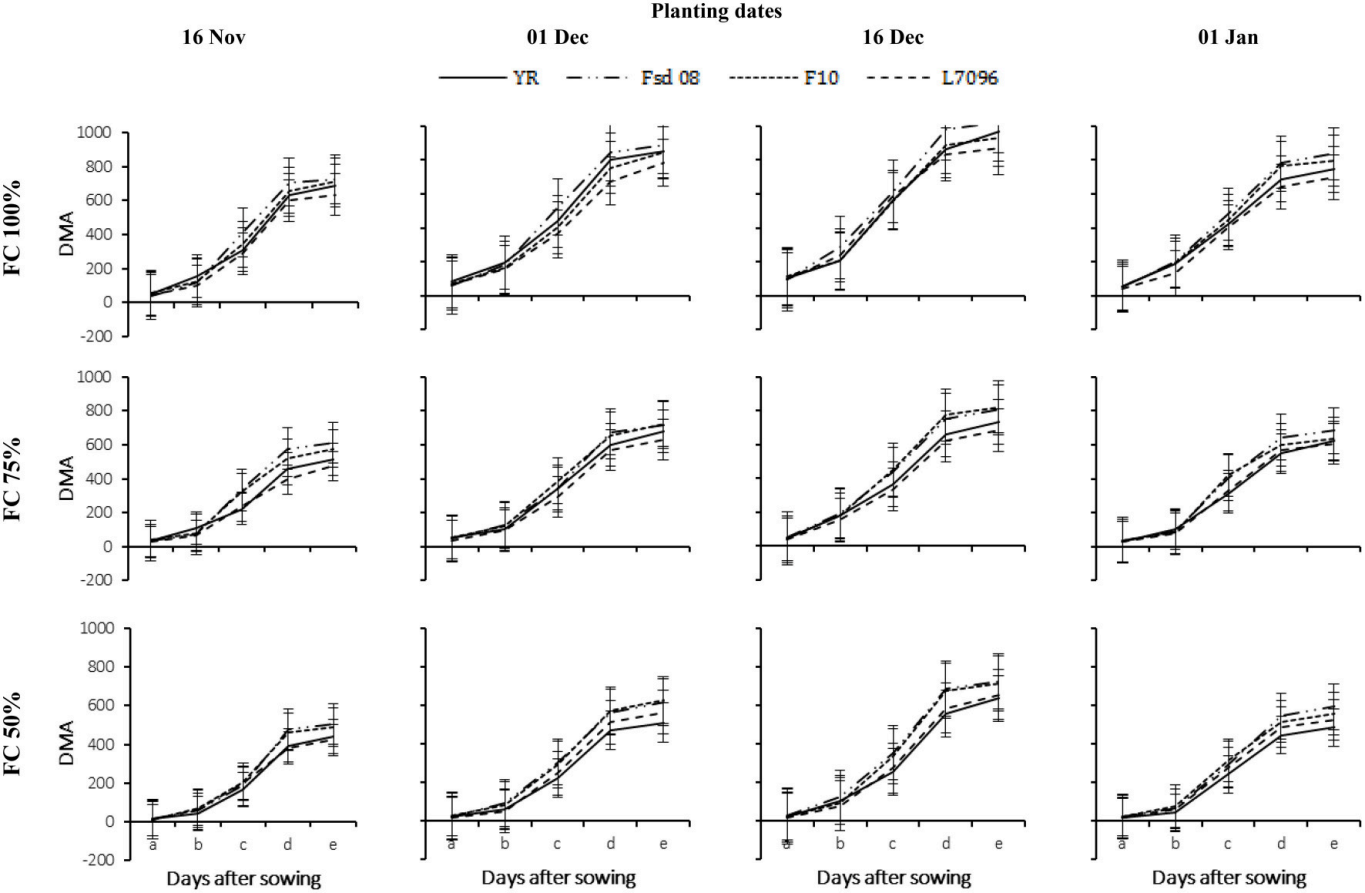
**Dry matter accumulation (DMA) of four wheat genotypes (G), viz., Yocoro Rojo (YR), Faisalabad 2008 (Fsd-08), FKAU-10 (F-10), and Galaxy L-7096 (L-7096) at three field capacities (FC) (100, 75, and 50%) and four planting dates (PD) (16 November; 01 December; 16 December, and 01 January) recorded at 15 days interval**. DMA was recorded from 4th weeks of sowing (2nd week of drought stress treatment application) at 30, 45, 60, 75, and 90 days after sowing (DAS) denoted by a, b, c, d, and e respectively. Vertical points are standard error bars representing statistically significant level of difference for DMA were recorded at 15 days interval for studied G, PD, and FC levels. Straight line, dotted dashed line, dotted line, and dashed line illustrate YR, Fsd-08, F-10, and L-7096 genotypes. Least significant difference (LSD) values among treatment means were 2.28, 10.45, 11.16, 11.22, and 4.87 for a, b, c, d, and e respectively.

**Figure 6:**
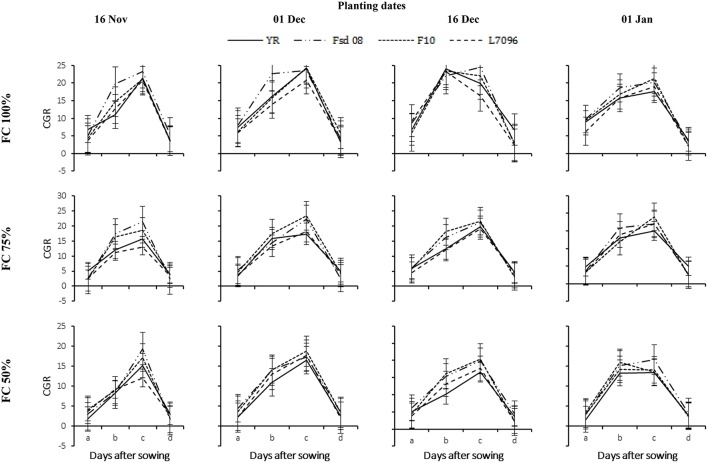
**Crop growth rate (CGR) of four wheat genotypes (G), viz., Yocoro Rojo (YR), Faisalabad 2008 (Fsd-08), FKAU-10 (F-10), and Galaxy L-7096 (L-7096) at three field capacities (FC) (100, 75, and 50%) and four planting dates (PD) (16 November; 01 December; 16 December, and 01 January) recorded at 15 days interval**. CGR was recorded from 4th weeks of sowing (2nd week of drought stress treatment application) at 30, 45, 60, 75, and 90 days after sowing (DAS) denoted by a, b, c, and d respectively. Vertical points are standard error bars representing statistically significant level of difference for CGR recorded at 15 days interval for studied G, PD, and FC levels. Straight line, dotted dashed line, dotted line, and dashed line illustrate YR, Fsd-08, F-10, and L-7096 genotypes. Least significant difference (LSD) values among treatment means were 0.49, 0.38, 0.81, and 0.29 for a, b, c, and d respectively.

**Figure 7:**
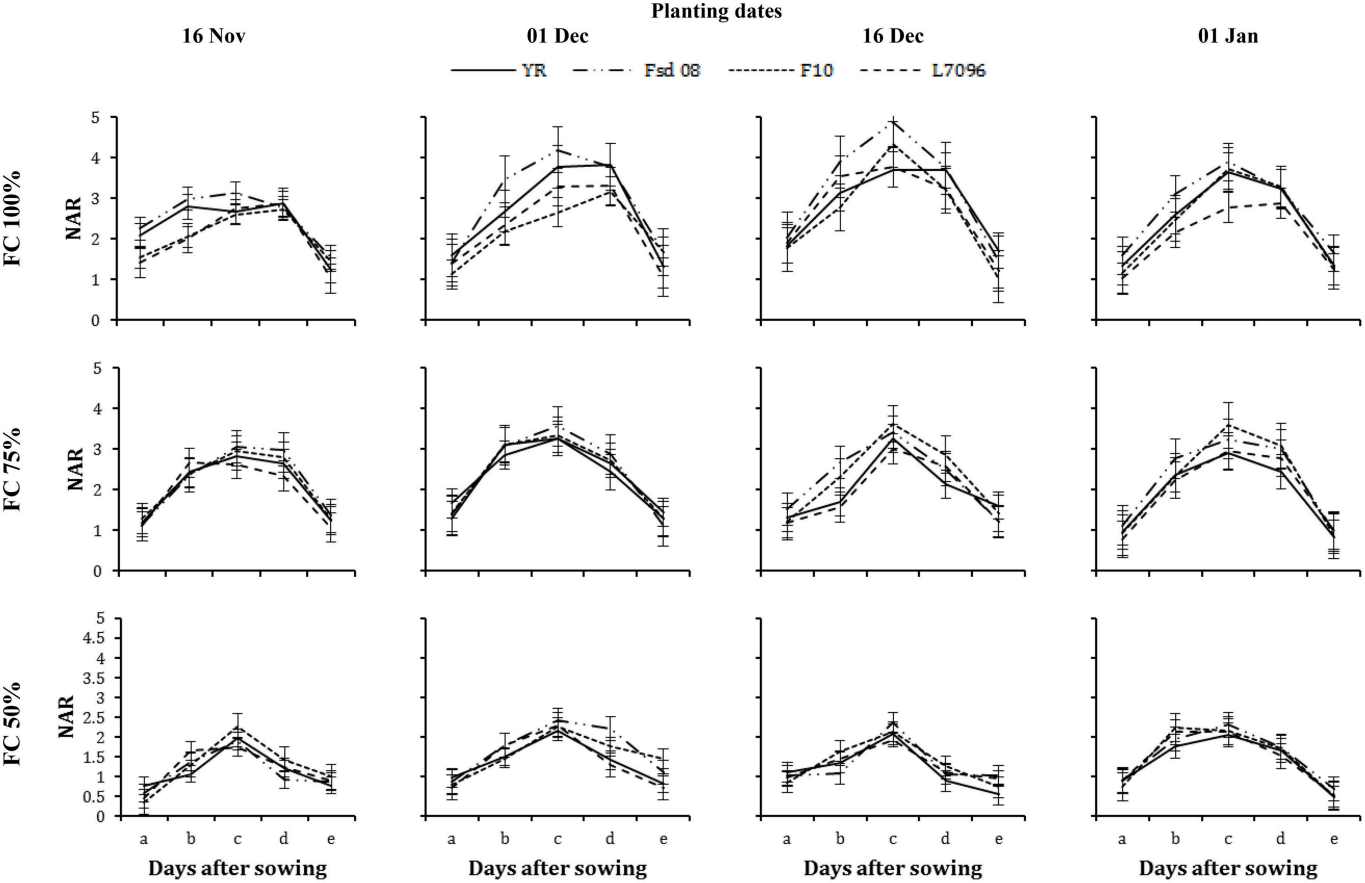
**Net assimilation rate (NAR) of four wheat genotypes (G), viz., Yocoro Rojo (YR), Faisalabad 2008 (Fsd-08), FKAU-10 (F-10), and Galaxy L-7096 (L-7096) at three field capacities (FC) (100, 75, 50%) and four planting dates (PD) (16 November; 01 December; 16 December, and 01 January) recorded at 15 days interval**. NAR was recorded from 4th weeks (2nd week of drought stress treatment application) at 30, 45, 60, 75, and 90 days after sowing (DAS) denoted by a, b, c, d, and e respectively. Vertical points are standard error bars representing statistically significant level of difference for NAR recorded at 15 days interval for studied G, PD, and FC levels. Straight line, dotted dashed line, dotted line, and dashed line illustrate YR, Fsd-08, F-10, and L-7096 genotypes. Least significant difference (LSD) values among treatment means were 0.04, 0.09, 0.19, 0.16, and 0.04 for a, b, c, d, and e respectively.

Growth of all wheat G gradually decreased with delay in sowing and severity of applied DS (Figures 3–**7**). The LAI gradually increased with the passage of time till 60 DAS and was decreased thereafter (Figure 3). At 90 DAS, declined sharply and the values were almost similar to LAI recorded at 30 DAS. Drought stress significantly diminished the LAI for all G. Averaged across G, DS at 75% FC and 50% FC reduced the LAI of November planted crop by 26–46%, December by 32–67%, and January by 07–40%, respectively, as compared with that under 100% FC. The LAI for wheat planted in November and January was 18% higher and 15% lower than the average for wheat planted in December. December planting (Dec 1 and Dec 16) favored all G for canopy establishment as indicated by their highest LAI values. Under severe DS, the maximum LAI of F-10 (5.29), Fsd-08 (5.10), L-7096 (4.29), and YR (4.08) were observed at 60 DAS, when wheat was planted in first week of December (Figure 3). The data regarding LAD also depicted similar trend as for LAI for all G but LAD continues to increase instead of sharp reduction as observed in LAI at 60 DAS (Figure 4). In December planted wheat, 50% FC recorded the least LAD value while 100% FC produced the highest LAD for all G. Under drought stress (75 and 50% FC), the highest LAD was observed for Fsd-08 (Figure 4).

Dry matter accumulation (DMA-g m^−2^) is the source for photosynthates translocation to sink during grain development. Drought stress severely hampered the DMA in all G; therefore, the highest values for DMA was recorded for non-stressed plots (Figure 5). Averaged across G and PD, DS at 75% FC and 50% FC decreased the DMA of wheat by 28 and 57%, respectively. The DMA increased with each crop phenological GS from tillering to maturity, nevertheless, the rate of DMA varied with different GS of the crop. Enhanced rate of DMA occurred from 45 until 75 DAS. At 100% FC, non-significant variations were observed among G regarding DMA. However, differences among G were apparent under DS, and Fsd-08 remained superior to all other studied G. The DMA in F-10 was statistically similar with Fsd-08, when crop was planted in mid-December. The minimum DMA was recorded in L-7096 (465 g m^−2^) at severe DS. The effect of PD was apparent under DS and December plantation resulted in higher DMA over November and January plantation at all FC levels.

Data regarding CGR and NAR varied significantly under the influence of various DS, G, and PD at each GS of the crop development (Figures 6, 7). The CGR progressively increased with the maximum value achieved at 60-75 DAS. At 60-75 DAS, the maximum and minimum CGR were recorded at 100% FC (24.46 g m^−2^ d^−1^) and 50% FC (20.43 g m^−2^ d^−1^) respectively, when wheat was sown in mid-December. However, at 75% FC, the maximum CGR (23.32 g m^−2^ d^−1^) was observed for wheat plants sown early in December. At 100% FC, all PD were statistically similar with each other regarding CGR. However, considerable reductions in CGR of all G were observed under DS. At 60-75 DAS, the CGR of G at 75 and 50% FC was 18 and 34% lower, respectively, as compared to 100% FC. Genotypes Fsd-08 and F-10 displayed greater tolerance to severe DS (50% FC) and recorded 17% higher CGR than local commercial variety YR.

The NAR increased gradually over time until 75 DAS, and declined sharply afterward as the crop reached maturity (Figure 7). Wheat planted in December had a greater NAR than when planted in November or January. The response of wheat G varied under the influence of various FC and PD for NAR. The highest NAR at 100% FC was found for Fsd-08 (4.87 g m^−2^ d^−1^), at 75% FC for F-10 (3.63 g m^−2^ d^−1^) and at 50% FC for Fsd-08 (2.43 g m^−2^ d^−1^), when wheat was planted at mid-December. The lowest NAR was measured for YR and L-7096 under severe DS for January planted crop. At 75% FC, F-10 documented higher NAR while under 50% FC; Fsd-08 performed better compared to all other G (Figure 7).

### Water use efficiency, grain yield, stress tolerance index, and relationship between crop growth indices and grain yield

The main effect of DS, PD, G and their two way and three interactions were significant (*p* ≤ 0.05) for final grain yield, water use efficiency and stress tolerance index except for main effect of PD for stress tolerance index (Table 5). Grain yield and stress tolerance index were superior for unstressed plots while water use efficiency was maximum for severe DS plots. The variations in crop GS and phenological development were translated into final grain yield. The maximum grain yield was attained for 100% FC and gradually declined for 75 and 50% FC. However, early planting and drought tolerant G mitigated the drought effects by producing significantly higher grain yield. Genotypes Fsd-08 and F-10 had 8-6% and 14-17% higher grain yield as compared to local G (YR) for unstressed and severely stressed plots respectively. Water use efficiency (9.21 kg ha^−1^ mm^−1^) and stress tolerance index (52%) were also significantly greater than the other three G. Plants under severe DS exhibited maximum water use efficiency, however; the stress tolerance index was lowest at this level. Earlier planting resulted in significantly greater water use efficiency but not stress tolerance index (Table 5).

**Table 5:** **Grain yield, water use efficiency, and stress tolerance index of wheat genotypes as influenced by different field capacities and planting dates under arid land conditions**.

**Treatments**	**Grain yield**	**Water use efficiency**	**Stress tolerance**
	**(kg ha^−1^)**	**(kg ha^−1^ mm^−1^)**	**index (%)**
**FIELD CAPACITY**			
100% FC	2953 a^*^	7.65 c	57.19 a
75% FC	2528 b	8.79 b	50.09 b
50% FC	1991 c	9.88 a	40.71 c
RLSD (*p* ≤ 0.05)	25.99	0.15	2.7
**PLANTING TIMES**			
16 Nov	2623 a	9.51 a	48.56
30 Nov	2532 b	8.55 c	51.36
16 Dec	2356 d	8.24 d	49.89
01 Jan	2451 c	8.8 b	47.51
RLSD (*p* ≤ 0.05)	20.42	0.12	ns
**GENOTYPES**			
YR	2434 c	8.54 c	49.03 c
Fsd-08	2619 a	9.21 a	52.29 a
F-10	2579 b	9.09 b	51.27 b
L-7096	2330 d	8.25 d	44.72 d
RLSD (*p* ≤ 0.05)	22.13	0.12	0.55

* *, Means with different letters are significant at p ≤ 0.05; ns, non-significant at p ≤ 0.05; YR, Yocoro rojo; Fsd-08, Faisalabad 2008; F-10, FKAU-10; L-7096, Galaxy L-7096; RLSD, Revised least significant difference*.

**Figure 8:**
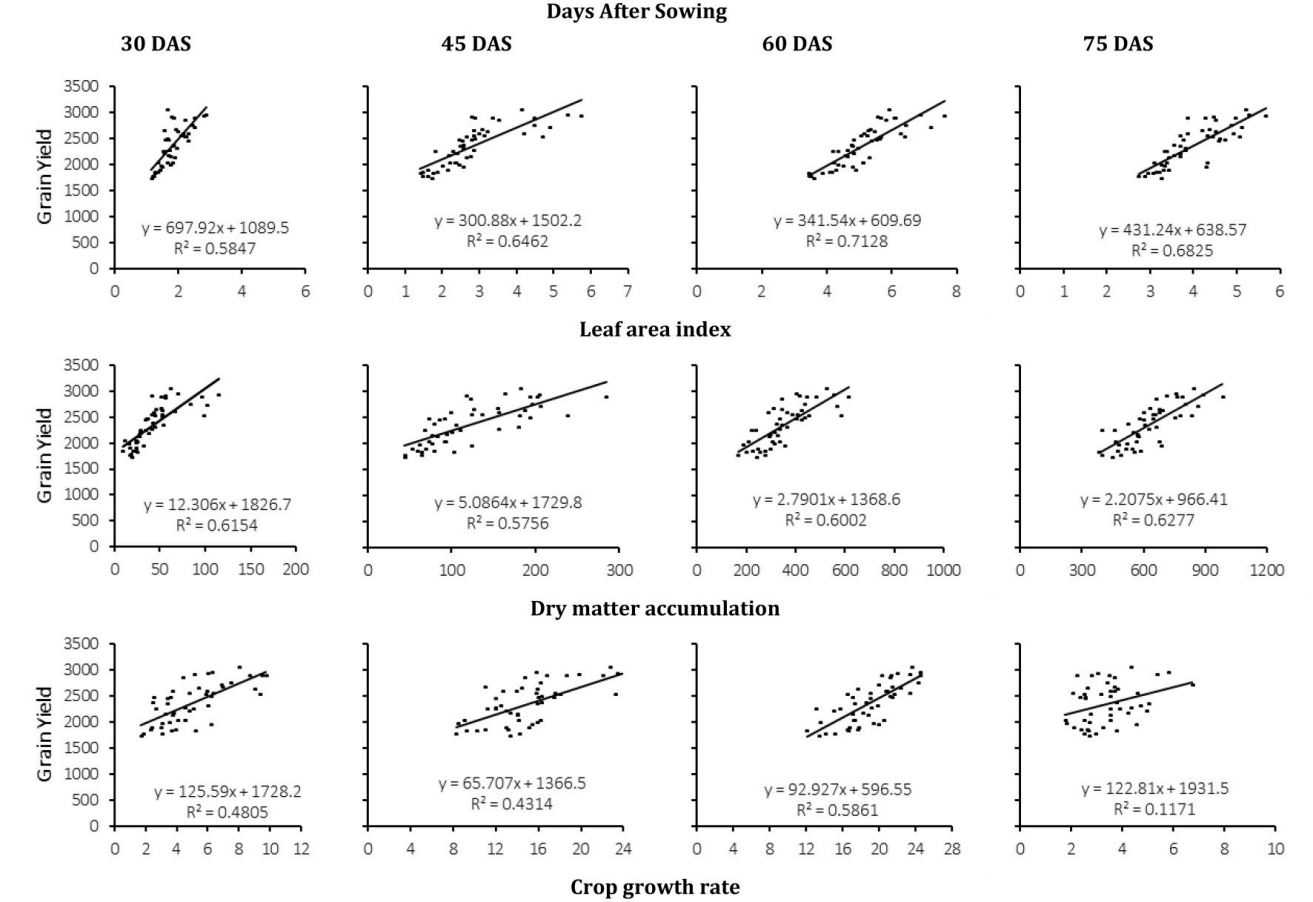
**Calibration of the regression model predicting wheat grain yield (kg ha^−1^) as a function of crop growth indices, leaf area index (LAI), dry matter accumulation (DMA g m^−1^), and crop growth rate (CGR g m^−2^ d^−1^) for pooled data of four wheat genotypes at three field capacities and four planting dates recorded at 15 days interval**. Data was evaluated for 30, 45, 60, and 75 days after sowing (DAS) and denoted by a, b, c, and d respectively. Y, x, and *R*^2^ are dependent variable, independent variable and Coefficient of determination respectively.

Regression analysis for the pooled data of DS, PD, and G for three crop growth indices (LAI, CGR, DMA) at four growth intervals 30, 45, 60, and 75 DAS was analyzed against grain yield using simple linear regression analysis (Figure 8). Grain yield dependency on crop growth indices was estimated along with trend line drawn across growth indices individually. All three growth indices were significantly and positively related to grain yield. The slope and strength of the relationships were the greatest at 60-75 DAS (Figure 8). Crop LAI had the strongest relationship with grain yield, overall.

## Discussion

A significant reduction in crop growth indices and time to complete phenological GS were observed for all G under DS and with delay in planting. This directly affected the G performance and adaptability to arid land conditions. These applied stresses affected each developmental stage i.e. germination, tillering, booting, heading, anthesis, and maturity. The enhanced negative effect of DS at later planting dates may be due to increased temperature stress at the grain filling stage (Figure 1). In our experiment, reduced plant height, biomass accumulation, days to maturity and grain filling period were the major stress susceptible traits that ultimately reduced final grain yield. These results are in line with the findings of Sial et al. (2005). Genotype F-10 had a longer vegetative growth period that resulted in a shorter reproductive phase under drought and late planting induced heat stress. Monasterio (2001) reported similar results regarding longer vegetative growth phase in comparison to grain filling duration for drought and heat stress under Mediterranean climate. Genotype Fsd-08 exhibited a prolonged grain filling period compared to YR which might be due to a different level of tolerance at each GS that leads to differential growth and grain yield (Araus et al., 2008; Tuberosa, 2012).

At moderate DS and late planting, inferior growth of some G might be correlated to decreased photosynthesis activity and carbohydrate translocation and assimilation (Spiertz and Vos, 1985). Under severe DS, plants may completely stop the translocation of carbohydrates to grains and shut down photosynthetic machinery, becoming totally dependent on stored sugars (Hall, 2000; Bita and Gerats, 2013). Zhang et al. (2006) reported that DS at jointing and anthesis stages reduced the grain yield of wheat by 14–25% as compared with well-watered crop. Climatic factors i.e., high temperature and low soil profile moisture content, negatively affected crop growth and development, especially DMA and CGR (Chakrabarti et al., 2011). Khan et al. (2012) proposed that the reason behind reduced growth and early flowering under severe heat stress may be due to sensitivity of metabolic processes that have limit the ability to effectively utilize available resources (Buck et al., 2007).

Variable behavior of G with changing stress levels and PD leads us to better understand the potential for tolerance and adaptability to local climate. Barnabás et al. (2008) argued that plants under severe DS attempted to survive by completing all GS within a short duration of time (Claeys and Inzé, 2013) as apparent from L-7096 growth habit at 50% FC in present study. It was further confirmed by Hossain and Da Silva (2012) who noticed that late sown wheat completed heading, grain filling and grain maturity stages by 23, 03, and 29 days earlier than early sown wheat due to the high temperature stress faced by the late sown wheat. Almost similar results were obtained in our study for January (late) sown wheat that faced elevated temperature (40°C) stress at grain filling stage. Genotypic responses to the same stresses can differ (Alghabari et al., 2014). Some G (highly sensitive to stress) even started to respond at germination and tillering stage by producing lower plant population and aborting initial tillers (Herbek and Lee, 2009). Plant canopy cover and flag leaf area have significant contribution to the grain filling stage (Khan et al., 2012; Ihsan et al., 2014). Severe DS and high temperature can cause considerable damage to plant canopy by scorching of leaves and sunburn of twigs and stems, leaf senescence and abscission, stunting primary growth leading to unfertile spikes, and smaller grain production, consequently limiting crop yield.

Wheat crops face DS throughout the world, but severity increases under Mediterranean climate. Plant response to stress varies depending upon its phenological GS (Farooq et al., 2009). Shortening of the grain filling period for F-10 and L-7096 under severe stress may be attributed to down regulation of enzyme activity (sucrose synthase, soluble starch synthase, or granule bound starch synthase) involved in signaling for starch accumulation during grain filling stage (Talukder et al., 2013; Liao et al., 2014). Genotypes with slower leaf senescence and higher sugar accumulation potential can limit stress effects by continuous photosynthesis and prolonging the grain filling period (Talukder et al., 2013). Dias et al. (2008) reported that heat stress above optimum level may cause grain shrinking in wheat through ultra-structural changes in aleurone layer and endosperm cell (Lidon and Dias, 2010; Farooq et al., 2011).

Regression analysis confirmed that yield was negatively correlated to growing degree days for early planting and days to complete vegetative GS. Improved crop growth indices were positively correlated to grain yield. Rapid grain filling rate was negatively correlated to economic yield as a shorter reproductive stage resulted in smaller grains as indicated by 1000-grain weight (Ihsan et al., 2014). There is general agreement that modern high-yielding cultivars are more adapted to favorable growing conditions, while landraces usually have higher yield under low input conditions. The findings of this study showed that breeders should choose stress tolerant G (e.g., Fsd-08 and F-10) based on stress severity in the target environment. The G that had longer periods at each GS and greater DMA also produced superior grain yield. Wheat phenological growth stages are useful indicators for wheat breeding. Genotypes with short stature, longer reproductive growth stage/staying green during reproductive stage and longer grain filling period are more promising under arid land conditions. Therefore, these evaluated wheat G using different phenological GS data can be exploited in breeding programs for terminal drought and heat stressed environments.

## Conclusions

This study demonstrated that drought stress and high temperature (late planting) induced severe negative effects on growth phenology and grain yield of wheat genotypes. Drought stress at 50% FC severely reduced plant height, as well as growth indices and forced all the genotypes toward early maturity. Heading and grain filling were the most sensitive growth stages to applied drought stress and delayed planting. Variation among genotypes was also apparent; Fsd-08 and F-10 demonstrated greater adaptability and tolerance to harsh arid-land environment while L-7096 was the most susceptible one. December planting of wheat mitigated the high temperature and drought induced adversities by providing optimal growth conditions to all genotypes, particularly Fsd-08 and F-10. Current research has significantly improved our understanding on wheat phenological development variations in response to drought, heat and planting dates. The findings of this work can be used to exploit exotic genotypes for adaptation to arid land conditions with limited input supplies. The prominent traits like higher tillering capacity, remaining green during the reproductive phase, longer grain filling period, and superior stress tolerance index may be of prime importance for breeders working on wheat stress tolerance under arid land conditions. In the short term, these exotic wheat genotypes seed can be imported and distributed to farmers to improve yield.

## Author contributions

MI and SI performed the field experiments. FE designed the experiment and did statistical analysis. MI and ID wrote the manuscript. SF did proof read the checked the manuscript for linguistic issues.

### Conflict of interest statement

The authors declare that the research was conducted in the absence of any commercial or financial relationships that could be construed as a potential conflict of interest.
